# Clavicular Osteomyelitis Secondary to Candida Parapsilosis Infection

**DOI:** 10.7759/cureus.8699

**Published:** 2020-06-19

**Authors:** Eukesh Ranjit, John Roxborough, Dean Davis, Amit Sapra, Priyanka Bhandari

**Affiliations:** 1 Family Medicine, Southern Illinois University School of Medicine, Springfield, USA; 2 Family and Community Medicine, Southern Illinois University School of Medicine, Springfield, USA; 3 Family Medicine, Decatur Memorial Hospital, Decatur, USA

**Keywords:** general practice, osteomyelitis, clavicular osteomyelitis, rare infection, family practice, fungal infection, debridement, candidal infection, candida parapsilosis, multidisciplinary team

## Abstract

Candida parapsilosis osteomyelitis is a rare diagnosis. Candidal infection can occur via hematogenous or local spread. A localized swelling around a bony structure should raise clinical suspicion. Diagnosis is made by a combination of imaging modalities and biopsy. Anecdotal case reports have been reported in medical literature and treatment guidelines are very limited. Treatment modality includes a combination of surgical debridement and antifungal therapy.

## Introduction

Osteomyelitis is an inflammation of the bone secondary to infection. It is an old disease that has existed for at least 291 million years, and the condition has been noted since the time of Hippocrates [1]. The majority of osteomyelitis cases are bacterial in etiology. Fungal osteomyelitis is rare, with Candida and Aspergillus being the common agents [2]. Osteomyelitis with other rarer fungi such as Blastomyces has been reported as well [3]. In cases of Candidal osteomyelitis, Candida albicans is the most common causative agent with Candida parapsilosis accounting for about 7% of the cases [4]. Management of Candidal osteomyelitis with surgical debridement and antifungal therapy is recommended by the Infectious Disease Society of America (IDSA) based on anecdotal case reports and open-label series [5]. Also, osteomyelitis of the clavicle is a rare type of infection and usually results from hematogenous or traumatic spread [6].

We present the case of a patient with multiple morbidities and history of sternal fracture that had been managed surgically three years prior to presentation. The patient presented with pain and swelling of the right upper chest, which was found to be caused by fungal clavicular infection with Candida parapsilosis. A multidisciplinary team approach was adopted to diagnose and manage the case and address the patient's needs.

## Case presentation

A 51-year-old male presented to the primary care clinic with complaints of pain along his collar bone. The patient had a past medical history of diabetes mellitus type II, morbid obesity (body mass index of 47.4), obstructive sleep apnea on bilevel positive airway pressure (BiPAP), chronic obstructive pulmonary disease (COPD), major depressive disorder, fibromyalgia, hepatitis C secondary to intravenous drug use (IVDU), polysubstance abuse on suboxone maintenance therapy, anxiety disorder, closed body fracture of the sternum, and sternal osteomyelitis.

Three years before the current presentation, the patient had developed sternal osteomyelitis following a closed body fracture of the sternum after stumbling and falling onto the corner of a dresser in the middle of the night. The patient had undergone sternal wound debridement by cardiothoracic surgery (CTS) and closure with re-advancement myocutaneous pectoralis flaps by plastic surgery at the time. Wound cultures grew strep viridans and a few diphtheroids, which had been treated with antibiotics.

**Figure 1:**
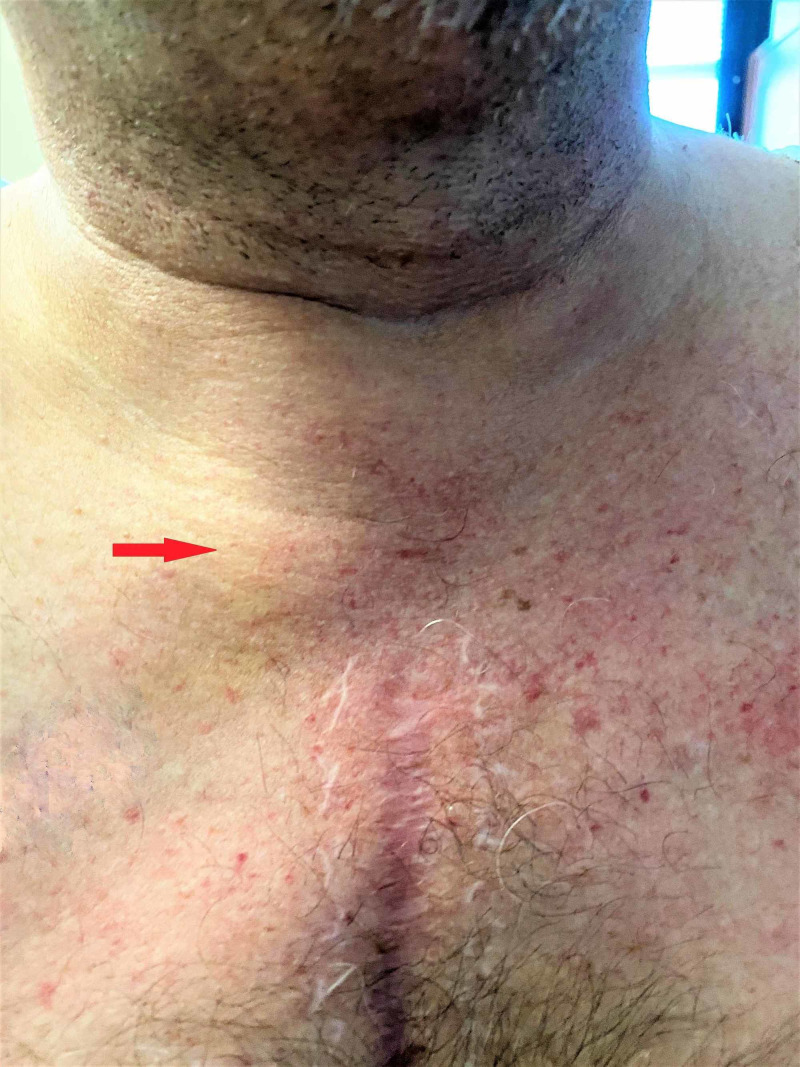
Diffuse swelling present around the medial end of the right clavicle (red arrow)

When the patient was initially seen in the clinic, he endorsed vague pain, swelling, and minimal tenderness along the right collar bone for about two weeks (Figure 1). No inciting event or trauma was reported.

**Figure 2:**
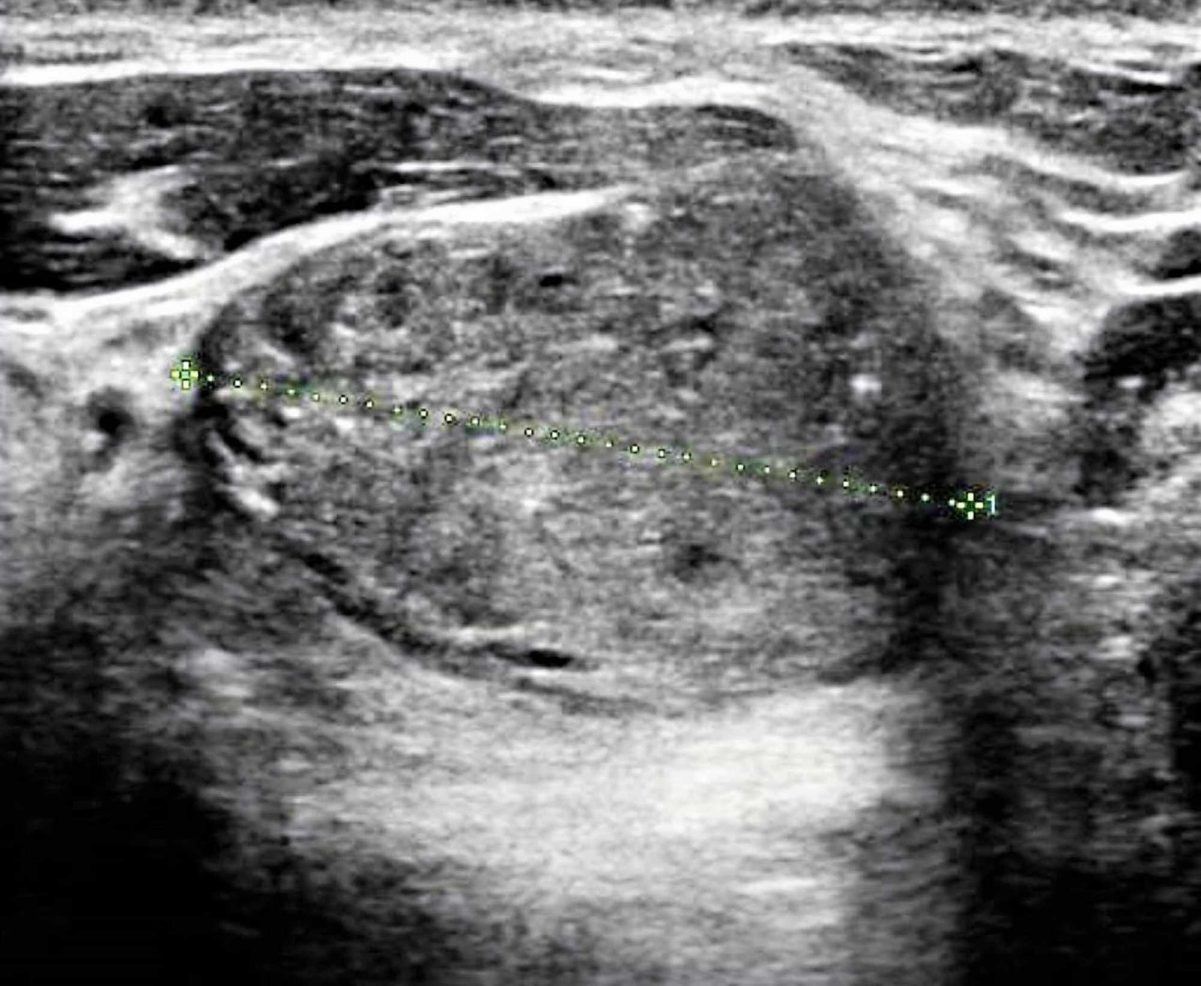
Ultrasound of the swelling shows a well-defined oval lesion around the medial end of the right clavicle (green dotted line)

Ultrasonography of the area was performed, which revealed a 5.1 X 3.2 X 2.7 cm heterogeneous soft tissue structure extending from the sternomanubrial joint (Figure 2).

**Figure 3:**
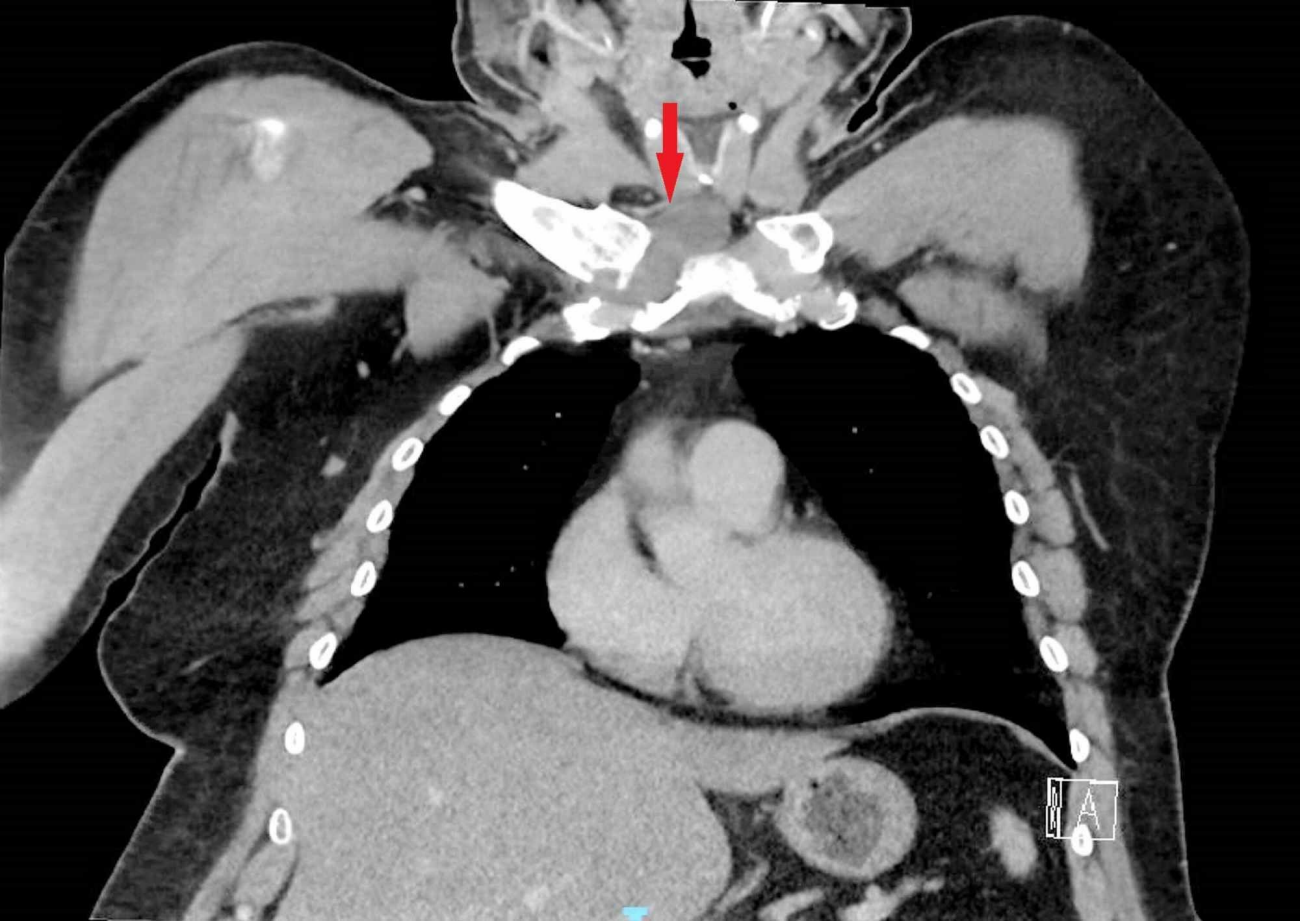
CT of the chest showing right sternoclavicular septic joint with osteomyelitis at the head of the right clavicle's connection with the manubrium (red arrow) CT: computed tomography

MRI of the chest was then ordered, which could not be completed due to insurance issues. An urgent outpatient CT of the chest was then ordered, which revealed right sternoclavicular septic joint with osteomyelitis at the head of the right clavicle's connection with the manubrium (Figure 3).

The patient's primary care provider (PCP) recommended that he be admitted for further management. During admission, WBC count was elevated at 10.9. Comprehensive metabolic panel (CMP) was unremarkable. Vancomycin and piperacillin/tazobactam were started empirically for osteomyelitis after blood cultures were drawn. Ultrasound-guided aspiration of the sternoclavicular joint was performed by the interventional radiology team, yielding 0.5 ccs of thick purulent fluid. Infectious disease (ID), CTS, and plastic surgery teams were consulted. The patient reported worsening anxiety secondary to hearing about COVID-19 cases in the news and left against medical advice (AMA).

**Figure 4:**
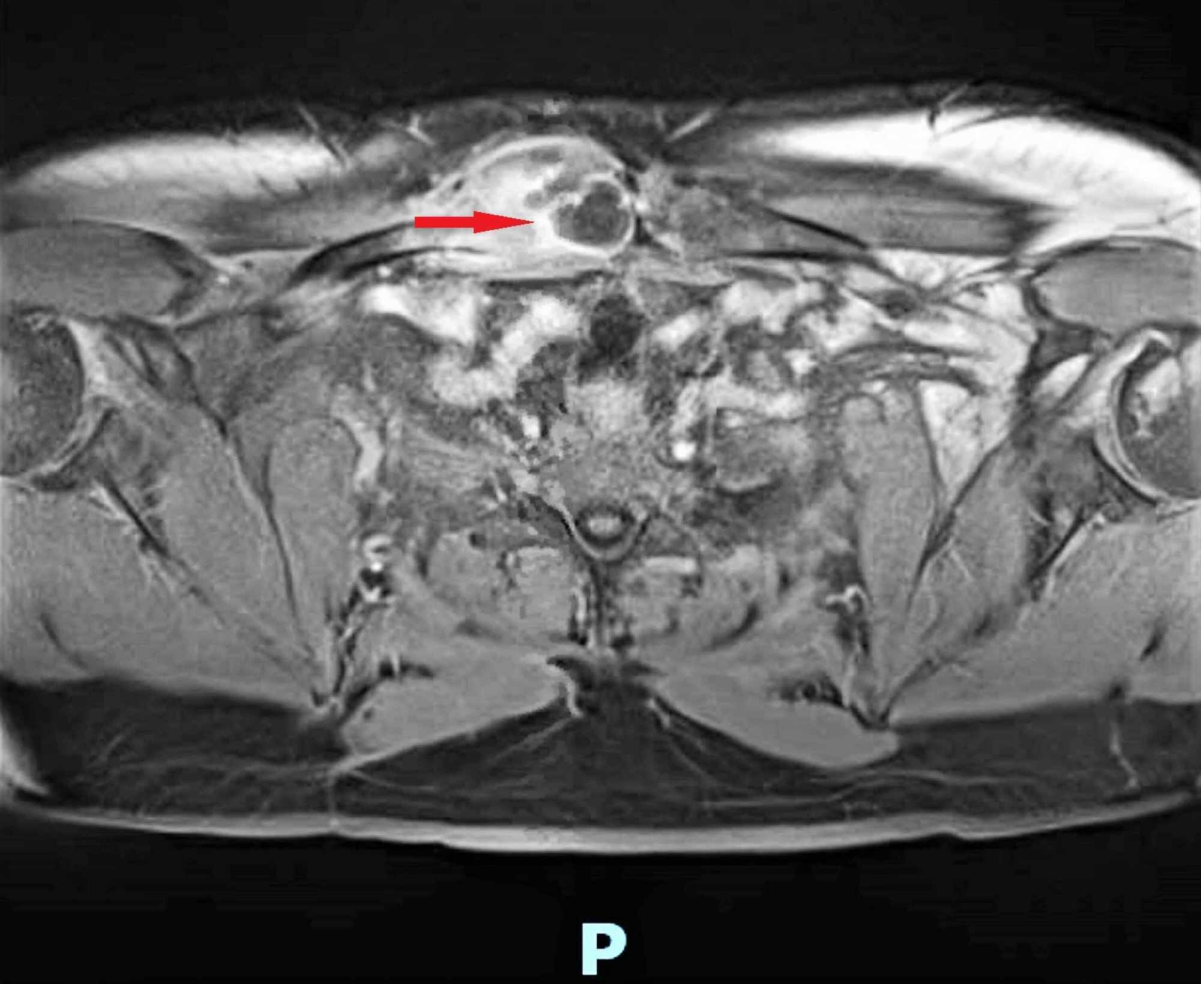
MRI of the right sternoclavicular joint showing right-sided sternoclavicular septic arthritis and osteomyelitis with associated periarticular abscess (red arrow) MRI: magnetic resonance imaging

With the coordination of care between the inpatient team and PCP, multiple telephone conversations were conducted by the PCP, which resulted in the patient returning to inpatient service six days after he left. Ultrasound-guided aspirate grew Candida parapsilosis. The patient was started on intravenous fluconazole, and antibiotic coverage with daptomycin and piperacillin/tazobactam was initiated. The patient reported worsening right clavicular pain at the time of readmission. WBC count was elevated at 13.5, and C-reactive protein (CRP) was elevated at 28.1. An MRI of the right sternoclavicular joint was performed, which revealed right-sided sternoclavicular septic arthritis and osteomyelitis with associated periarticular abscess (Figure 4).

Open incision and drainage of the right sternoclavicular joint was performed by the CTS team, followed by sternal wound closure by the plastic surgery team. Sternoclavicular joint aspirate culture revealed Candida parapsilosis again, and a subclavicular tissue culture revealed Staphylococcus aureus. Intravenous fluconazole was continued for a week, after which it was transitioned to oral fluconazole. Intravenous daptomycin was continued at the time of discharge for eight more weeks.

## Discussion

Candida parapsilosis, formerly known as Monilia parapsilosis, is found in nature and as a human skin commensal organism. Initially isolated in 1928 from a patient with diarrhea in Peurto Rico, it was thought to be nonpathogenic. However, in 1940, this pathogen was found to be associated with endocarditis/sepsis in a patient with a history of IVDU [7].

When considering osteomyelitis, the differential diagnosis includes common pathogens such as Staphylococcus aureus. If the patient has certain risk factors such as sickle cell anemia or thalassemia, Salmonella species are also considered [8]. In the cases of patients similar to the one discussed here, with a history of IVDU, diabetes, morbid obesity, tobacco use, and previously closed injury resulting in osteomyelitis, the clinician should be aware of the increased risk of osteomyelitis.

Yingling et al. reported a case of Candida parapsilosis in an immunocompromised HIV patient with a history of hardware implantation [9]. Although the patient was not HIV-positive, he did have underlying health conditions and had undergone treatments associated with fungal osteomyelitis and diabetes, and had a history of IVDU and broad-spectrum antibiotic use [10]. Other common risk factors for fungal infections, such as total parenteral nutrition and central venous catheters, were not present in this case. Our case emphasizes the importance of maintaining a broad differential when assessing patients with septic arthritis/osteomyelitis. Gamaletsou et al. noted that although fungal osteomyelitis remains rare, as many as 10% of rib osteomyelitis cases were due to fungal pathogens, and the incidence is increasing with the increasing number of susceptible hosts [11,12].

Treatment strategies for Candida parapsilosis osteomyelitis incorporate surgical debridement with antifungal chemotherapy [4]. Both Candida albicans and parapsilosis are biofilm-producing pathogens, thus necessitating the debridement of the infected tissues to improve the chances of resolution [9]. Our patient was treated with extensive surgical debridement, intravenous daptomycin, and intravenous fluconazole as an inpatient and was eventually transitioned to oral fluconazole for outpatient treatment. At the time of this writing, he is continuing with the treatment.

Given the increasing prevalence of Candida parapsilosis osteomyelitis, it is prudent for the family physician to understand this pathogen, commonly associated risk factors, and management techniques [10]. Our patient's case showcases the need for family physicians to be aware of Candida species as a pathogen, particularly in higher-risk patients frequently encountered in our routine practice.

## Conclusions

Candida parapsilosis involving clavicle is a rare form of osteomyelitis. The patient may present with vague swelling and discomfort on the upper chest. Diagnosis involves initial imaging followed by a biopsy, and management includes surgical debridement and antifungal therapy. Management with a multidisciplinary team approach is necessary. Potential anxiety related to numerous procedures and prolonged course of antibiotics should be addressed. Proper communication and transition of care between the inpatient team and PCP helps with issues of patient non-compliance and provides a better outcome for the patient.
